# Metastatic Gallbladder Carcinoma to the Ovary Presenting As Small Bowel Obstruction

**DOI:** 10.7759/cureus.46147

**Published:** 2023-09-28

**Authors:** Sara Salehiazar, Komeil Mirzaei Baboli, Joshua Lee, Valerie Ann Dib, Caroline Yap, Laron McPhaul

**Affiliations:** 1 Pathology and Laboratory Medicine, Harbor-University of California, Los Angeles (UCLA) Medical Center, Torrance, USA; 2 Radiology, Harbor-University of California, Los Angeles (UCLA) Medical Center, Torrance, USA

**Keywords:** metastatic adenocarcinoma of gallbladder, rare tumors, ovarian mass, ovarian tumor, metastatic gall bladder

## Abstract

The ovaries are a common site of metastasis from different organs, especially from the gastrointestinal tract. However, metastasis from the gallbladder to the ovaries is very rare. Our case involves a 57-year-old female who presented with abdominal pain and distension. Radiologic imaging suggests the possibility of a primary ovarian tumor causing small bowel obstruction. Grossly, the cystic mass in the right ovary closely resembles the typical characteristics of a primary ovarian tumor. Histologic examination revealed adenocarcinoma. Positive immunostaining for CA 19-9, cytokeratin-7 (CK7), CEA, and CDX2 and negative reaction to CK20, PAX8, and CA 125 are compatible with a pancreaticobiliary/gallbladder origin. Considering the results obtained from imaging, which included gallbladder wall thickening and the presence of a mass within the gallbladder, alongside the pancreas appearing normal, the primary site of concern was determined to be the gallbladder. It is important to consider primary sites other than the ovary in the presence of previous or concurrent lesions elsewhere, such as gastrointestinal (GI) or pancreaticobiliary tract since treatment regimens are different. Clinicoradiologic correlation and immunohistochemistry aid in differentiating this secondary ovarian tumor from a primary ovarian carcinoma.

## Introduction

Carcinoma of the gallbladder is an incidental finding in 1% of patients undergoing cholecystectomy due to presenting cholelithiasis [1]. Gallbladder cancer is a rare and aggressive malignancy accounting for 1.2% of all global cancer diagnoses [2]. Most patients are diagnosed at a more advanced stage with poor clinical outcomes due to the absence of symptoms or non-specific clinical presentation and lack of effective screening diagnostic methods. Liver and regional lymph nodes are the most common sites for metastasis. Secondary ovarian tumors comprise 10-25% of all ovarian malignancies; most common sites of origin include gynecologic (breast, endometrium) and non-gynecologic organs, especially the gastrointestinal tract (stomach, appendix, colorectal); Krukenberg tumor represents ovarian metastasis of signet ring cell adenocarcinoma primarily from the stomach and less commonly, colorectal, breast and appendix [3]. Metastasis to the ovary may precede the detection of the primary gallbladder tumor [3]. Metastasis from the gallbladder to the ovaries is rare, and only a few reports are available [4]. Some of these were initially misdiagnosed as primary ovarian tumors. Paucity of cases and limited data have contributed to their poor recognition. As gallbladder carcinoma can also have a mucinous histology subtype, differentiating it from primary ovarian cancer becomes a diagnostic challenge when synchronous tumors are present [5]. It is crucial to identify the unusual spread of gallbladder adenocarcinoma to the ovary. In this study, we present a very rare case of metastatic gallbladder adenocarcinoma to the ovary, with diagnosis reached using clinicoradiologic correlation and immunohistochemical characterization.

## Case presentation

**Figure 1:**
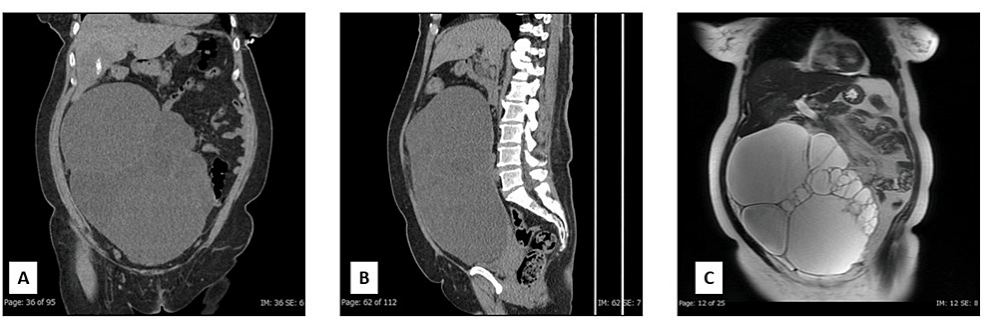
Abdominal CT scan, whole-body PET scan, and MRI of the patient. (A) Coronal image of the abdomen and pelvis without contrast demonstrates a complex multiseptated cystic mass of up to 24.6 x 18.1 x 11 cm arising from the right adnexa. An ill-defined 7.1 x 5.5 x 5.1 cm low-density lesion in the right hepatic lobe shows central calcifications. (B) A sagittal image of the abdomen and pelvis without contrast redemonstrates a complex multiseptated cyst mass, 24.6 cm in craniocaudal dimension and arising from the right adnexa. (C) Coronal T2 Single-Shot Fast Spin Echo (SSFSE) ARC image of the abdomen and pelvis shows a large 26 x 19 x 13 cm multiloculated cystic structure that appears to originate from the right adnexal region and demonstrates T2 hyperintense signal with multiple thickened septations.

The patient is a 57-year-old postmenopausal multiparous Hispanic female with no significant past medical or family history. She presented with 6.5 kg of unintentional weight loss, decreased appetite, nausea, vomiting, abdominal pain, obstipation, and abdominal distention. Physical examination was remarkable for a large, firm, non-mobile mass in the hypogastric region extending to the right iliac fossa. Initial laboratory tests showed increased serum CA 125 (36.5 U/mL) and CA 19-9 (1315 U/mL) and normal liver function tests (serum alanine transaminase {ALT}, aspartate transaminase {AST}, alkaline phosphatase {ALP}), serum carcinoembryonic antigen (CEA), and coagulation and kidney function tests. Abdominal CT scan, whole-body PET scan, and MRI revealed a massive multilocular right ovarian cystic mass measuring approximately 30 cm and causing closed loop bowel obstruction of the ileum (Figures 1A-1C).

**Figure 2:**
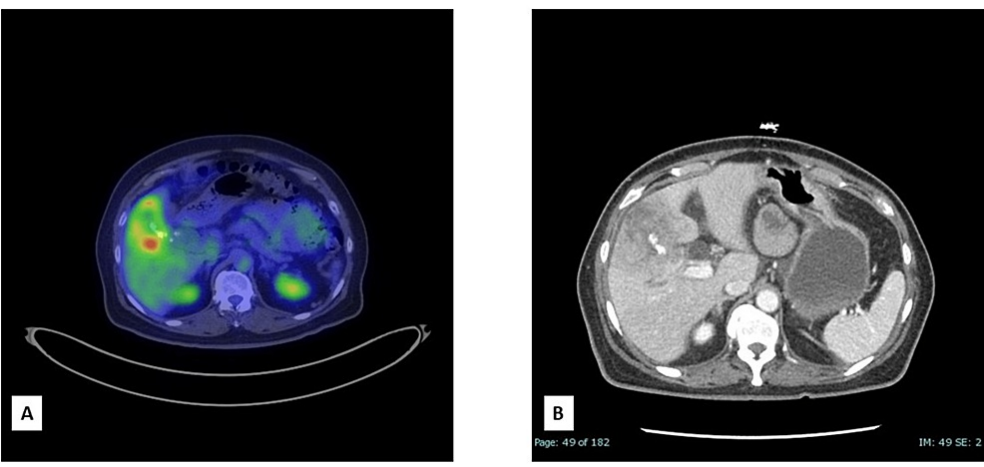
PET-CT and axial contrast-enhanced image of the patient. (A) Axial image from a fluorodeoxyglucose (FDG) PET-CT examination shows a 6.8 x 2.9 cm (AP x transverse) ill-defined low attenuation mass in the inferior liver with internal calcifications and effacement of the gallbladder wall. This mass demonstrates increased FDG activity with a standardized uptake value (SUV) max of 9. (B) An axial contrast-enhanced image of the abdomen redemonstrates irregular gallbladder wall thickening with low attenuation in the surrounding hepatic parenchyma, favored to represent mass invasion. Calcifications within the abnormal gallbladder may represent gallstones. Dilatation of the common bile duct and partially visualized right intrahepatic bile duct dilatation are also observed.

PET-CT revealed diffuse thickening of the gallbladder wall and a gallbladder mass invading the inferior right hepatic lobe, raising concern for biliary malignancy (Figures 2A, 2B). CT-guided needle core biopsy of the low-attenuation region in the liver revealed a moderately differentiated adenocarcinoma with immunophenotypic features (CK7, CK17, CK19, CDX2, IMP4 positive, and CK20, PAX8, BRST2, GATA3, glypican 3, and arginase negative) consistent with biliary or pancreatic origin.

**Figure 3:**
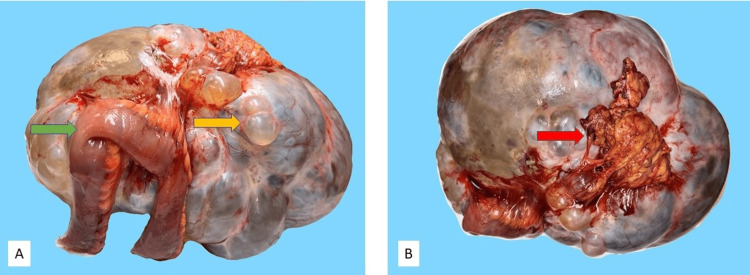
Patient's ovarian cystic mass. (A) Large ovarian cystic mass shows lobulated, mostly glistering outer surface with multiple clear fluid-filled cysts (yellow arrow) and has an adherent, focally compressed, and distorted portion of small bowel (green arrow). (B) Peritoneal fat is focally adherent to the cystic mass (red arrow).

The patient underwent exploratory laparotomy two weeks later and was found to have copious ascites, a large right ovarian multicystic mass that filled the entire abdomen, a 10 cm portion of small bowel adherent to the mass, and a palpable hard area over the liver and gallbladder consistent with gallbladder malignancy. Peritoneal fluid (ascites) collection and peritoneal washing for cytology and right salpingo-oophorectomy with en bloc small bowel resection were performed. Peritoneal fluid cytology was positive for metastatic adenocarcinoma. Gross examination showed a 5016 g aggregate of a 32 x 25 x 12 cm large multilobulated cystic ovarian mass attached to a 9 cm long, 1.5 cm in maximum diameter fimbriated fallopian tube, a 19 cm long, up to 2.8 cm in diameter small bowel segment, small amount of tan-yellow peritoneal fat, and mesenteric fat containing two lymph nodes. The cystic mass displayed multilobulated tan-pink outer surface with scattered areas of dusky blue-gray discoloration (Figures 3A, 3B). Opening of the cysts yielded copious tan-brown fluid with some semisolid tan-brown paste-like material. The cystic inner surface was multiloculated with no papillary projections.

**Figure 4:**
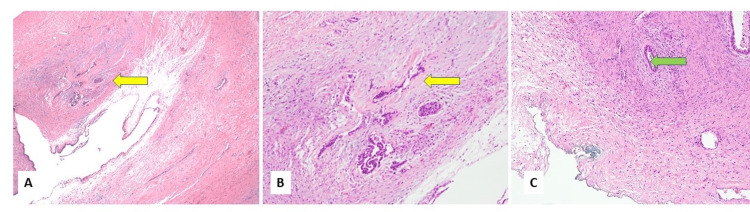
Histological examination of the cyst. (A and B) Small foci of atypical glands show morphology resembling pancreaticobiliary carcinoma (yellow arrow). (C) Tumor glands (green arrow) invade the ovarian stroma near the blue-inked ovarian surface.

Histologic examination revealed multiple cystic spaces lined by cuboidal to columnar epithelium and filled with acellular eosinophilic material. Seven of 32 sections of the cystic mass showed scattered small foci of atypical glands resembling those of pancreaticobiliary carcinoma (Figures 4A, 4B). These neoplastic acini showed haphazard arrangement within and infiltration of the ovarian stroma near the ovarian surface (Figure 4C).

**Figure 5:**
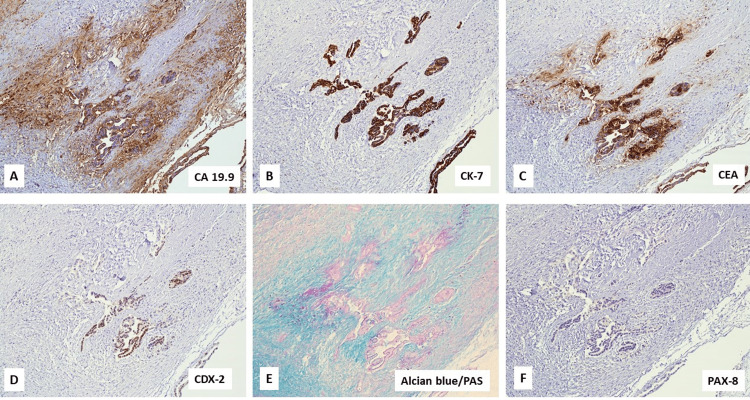
Tumor cells are strongly positive for CA 19-9 (A), CK-7 (B), CEA (C), and CDX2 (D), positive for Alcian blue-PAS (E), and negative for PAX8 (F). PAS: Periodic Acid-Schiff; CEA: carcinoembryonic antigen

The tumor cells showed positive staining for CA 19-9, CK7 (intense, cytoplasmic), CEA (strong), CDX2 (diffuse, nuclear), and negative reaction to CK20, CA 125, and PAX8, and positive Alcian blue/PAS staining (Figures 5A-5F).

**Figure 6:**
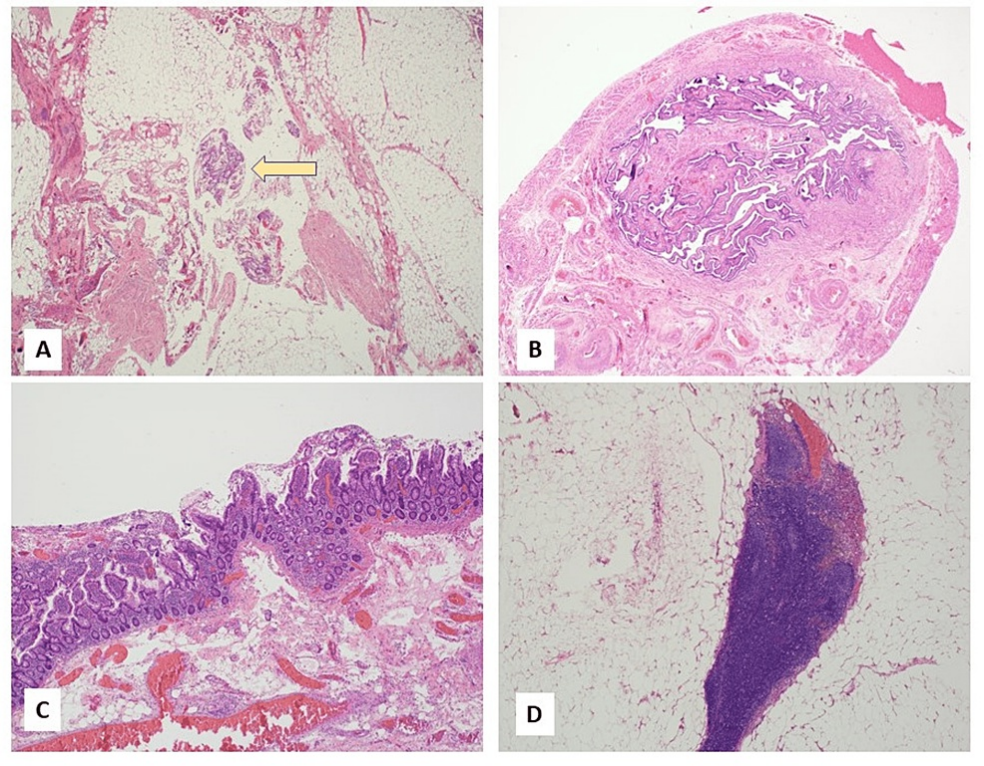
Additional histological findings. The images show (A) microscopic focus of metastatic adenocarcinoma in the peritoneal fat (yellow arrow), (B) unremarkable fallopian tube, (C) an unremarkable portion of small bowel, and (D) benign lymph node free of tumor.

Within the peritoneal fat were small foci of malignant glands with morphologic and immunostaining features similar to the ovarian cystic mass (Figure 6A). Mesenteric fat attached to the small bowel shows a small focus of metastatic adenocarcinoma. The attached right fallopian tube (Figure 6B), small bowel (Figure 6C), and two lymph nodes were otherwise unremarkable and free of tumor (Figure 6D). Mismatch repair (MMR) studies showed intact expression of MLH1, MSH2, MSH6, and PMS2, unlikely for Lynch syndrome.

## Discussion

Differentiating between primary and metastatic ovarian tumors can commonly pose a dilemma for pathologists and clinicians. It is crucial to make an accurate diagnosis as treatment plans definitely differ. Gallbladder carcinoma is discovered in about 1% of patients undergoing cholecystectomy, with adenocarcinoma accounting for 70-90% of gallbladder cancers [1]. However, different studies have indicated that only a small percentage of patients with gallbladder carcinoma will ever develop metastasis to the ovary [6]. The pathophysiology of how gastrointestinal cancer can metastasize to the ovary remains unknown. Interestingly, the laterality of the involved ovary is not related to that of the primary tumor [6]. Most metastatic ovarian neoplasms tend to be bilateral, in contrast to unilateral primary ovarian neoplasms [7].

Imaging characteristics of gallbladder carcinomas with metastasis to the ovary may resemble cholecystitis or cholelithiasis, and reports are rare [8]. The majority of patients present with non-specific abdominal or pelvic symptoms. During surgery, the presence of abnormal findings involving the surface of the gallbladder with multilobulated growth patterns around the primary site (gallbladder) should raise suspicion for a non-ovarian primary tumor.

Most primary ovarian mucinous tumors are unilateral, large, multilocular, smooth-surfaced, and expansile. Most metastatic ovarian tumors are bilateral, smaller, multinodular, infiltrative, and with frequent ovarian surface involvement. Tumors from pancreaticobiliary tract origin can grossly resemble primary ovarian malignancies, as the case in this study. Multicystic areas filled with mucin and areas of hemorrhage and necrosis can be present. Microscopic diagnosis can sometimes be challenging, mainly due to the bland epithelial lining of the tumor glands. Awareness of this possibility and consideration of clinical findings, gross characteristics, histologic findings, and immunohistochemistry may help avoid this diagnostic pitfall. It is important to note that in limited samples, especially during frozen section evaluation, this can be a particularly treacherous area.

Microscopic examination of our case revealed infiltration of variably sized and spaced neoplastic glands with apical pale mucinous material. These glands were positive for CK7, CDX2, and CA 19-9, and negative for CK20, CA 125, and PAX8, favoring a diagnosis of metastatic adenocarcinoma of pancreaticobiliary origin. In contrast, primary ovarian tumors are usually large, multicystic masses with smooth surfaces, seldom infiltrative histology, nuclear expression of PAX8, and dual positivity for CK7 and CK20 [8].

Management and prognosis of primary ovarian cancer differ from that of metastatic ovarian cancer from gallbladder primary cancer. Surgical management is useful for primary ovarian cancers and is limited to early-stage gallbladder cancers. Chemotherapy regimens are also different. The five-year survival rate of primary gallbladder cancer is abysmal (5%), whereas that of stage IV ovarian cancer is 19% and stage III ovarian cancer is 40-50% [8]. The combination of surgery with chemotherapy is associated with a significant improvement in survival in stage IV gallbladder cancer patients compared to patients treated with either surgery or chemotherapy alone [9].

## Conclusions

Metastatic gallbladder carcinoma should be considered in the differential diagnosis of ovarian masses, especially when imaging shows any abnormality in the gallbladder such as calcification or thickening of the gallbladder wall. Diagnosis can be challenging especially in mucinous tumors because imaging and gross characteristics can be similar in primary and metastatic ovarian malignancies. Accurate diagnosis can be achieved through detailed clinical history, radiologic studies, and careful evaluation of histology and immunohistochemistry. The management of metastatic gallbladder adenocarcinoma differs from that of primary ovarian carcinoma. Given the rarity of this diagnosis (ovarian carcinoma originating from the primary gallbladder carcinoma), which is now receiving increased recognition and is more likely to be documented, there is a need for the establishment of guidelines and diagnostic criteria.
